# Effects of Italian Mediterranean Organic Diet on the Gut Microbiota: A Pilot Comparative Study with Conventional Products and Free Diet

**DOI:** 10.3390/microorganisms13071694

**Published:** 2025-07-18

**Authors:** Laura Di Renzo, Giulia Frank, Barbara Pala, Rossella Cianci, Giada La Placa, Glauco Raffaelli, Roselisa Palma, Daniele Peluso, Antonino De Lorenzo, Paola Gualtieri, on behalf of Clinical Nutrition and Nutrigenomics Project Group

**Affiliations:** 1 Section of Clinical Nutrition and Nutrigenomics, Department of Biomedicine and Prevention, Tor Vergata University of Rome, Via Montpellier 1, 00133 Rome, Italy; laura.di.renzo@uniroma2.it (L.D.R.); delorenzo@uniroma2.it (A.D.L.); paola.gualtieri@uniroma2.it (P.G.); 2 PhD School of Applied Medical-Surgical Sciences, Tor Vergata University of Rome, Via Montpellier 1, 00133 Rome, Italy; giulia.frank@ymail.com (G.F.); laplacagiada@gmail.com (G.L.P.); glauco.raffaelli@yahoo.it (G.R.); roselisa.palma@gmail.com (R.P.); daniele.peluso@uniroma2.it (D.P.); 3 School of Specialization in Food Science, Tor Vergata University of Rome, 00133 Rome, Italy; 4 Department of Translational Medicine and Surgery, Catholic University of the Sacred Heart, 00168 Rome, Italy; rossella.cianci@unicatt.it; 5 Fondazione Policlinico Universitario A. Gemelli, Istituto Di Ricovero E Cura a Carattere Scientifico (IRCCS), Largo Agostino Gemelli 8, 00168 Rome, Italy

**Keywords:** mediterranean diet, organic food, gut microbiota, exposome, body composition

## Abstract

The human exposome, including dietary exposures such as pesticides, additives, and environmental contaminants, plays a critical role in shaping the gut microbiota (GM) and long-term health outcomes. While the Mediterranean Diet is known for its health-promoting effects, the role of food quality, specifically organic vs. conventional products, in modulating GM within this dietary pattern remains underexplored. The aim of this study was to evaluate (1) whether an Italian Mediterranean Organic Diet (IMOD) confers additional benefits compared to an Italian Mediterranean non-Organic Diet (IMnOD), and (2) the impact of IMOD and IMnOD versus a free diet (No Diet) on GM and anthropometric parameters. A randomized, controlled trial was conducted on 39 healthy subjects. Eligible subjects were divided into the following groups: (1) 4 weeks No Diet, (2) 4 weeks IMOD, and (3) 4 weeks IMnOD. Microbiota profiling (16S rRNA sequencing), body composition (BIA), and dietary adherence (MEDAS, FFQ) were evaluated. Distinct microbial shifts following both IMOD and IMnOD compared to No Diet were revealed. Several taxa previously associated with short-chain fatty acid (SCFA) biosynthesis (i.e., *Anaerobutyricum hallii*, *Anaerostipes hadrus*, and *Dorea longicatena*) were increased after both Mediterranean Diet interventions, while *Parabacteroides distasonis* showed a specific increase in the IMOD group. No significant changes in body weight or composition were observed. These findings suggest that adherence to a Mediterranean Diet, regardless of food source, reshapes the gut microbiota, while organic food intake may influence specific microbial trajectories. Our results support the relevance of food quality in dietary interventions.

## 1. Introduction

According to the exposome (the set of environmental and internal factors that interact with human health), diet represents one of the main pathways of exposure to potentially harmful environmental xenobiotics, such as pesticides, plasticizers, and heavy metals [1]. Recent advances have emphasized the bidirectional relationship between the exposome and the gut microbiota (GM), underscoring how the GM serves both as a sensor and a modulator of environmental exposures [2]. Indeed, while the GM is highly sensitive to environmental exposures such as diet, pesticides, and xenobiotics, core components of the exposome, it can also modulate the host’s exposome through metabolic and immunological feedback mechanisms. Notably, microbial communities influence the transformation, activation, or detoxification of exogenous compounds, thus modifying the host’s internal exposure profile. Moreover, dysbiosis-associated pathophysiological states (e.g., increased intestinal permeability, chronic inflammation) may alter the host’s susceptibility to environmental agents, effectively shaping the exposome in return [2]. The intestinal microbiota is highly sensitive to xenobiotics that can alter its composition, reduce diversity, and impair its metabolic function. Conversely, the GM can actively transform these compounds, generating metabolites that may be more or less toxic depending on the host–microbiota interactions. This dynamic interplay affects immune regulation, intestinal barrier function, and systemic homeostasis, reinforcing the notion that diet-mediated exposure to environmental contaminants plays a pivotal role in shaping microbial–host relationships [2].

Recent metabolomic and metagenomic studies have shown that the chronic ingestion of pesticide residues can significantly alter the GM profile, reducing biodiversity, inducing dysbiosis, and promoting altered inflammatory and metabolic phenotypes [3,4,5,6].

In particular, pesticide-induced microbial shifts have been associated with changes in fecal and urinary metabolites, supporting the hypothesis that GM may act both as a target and a modulator of chemical toxicity [7]. The intake of organic products measurably reduces the impact of pesticides, supporting the prevention of chronic diseases by reducing the cumulative toxic load [8]. Several studies have reported that organic food intake can have a positive impact on the gut ecosystem, regardless of the nutritional content alone [9].

The GM is essential for human health, as it helps regulate and preserve the integrity of the intestinal barrier, protects against the intrusion of harmful microbes, supports the synthesis and digestion of nutrients, and enhances the development of both the innate and adaptive immune systems [10].

The absence of pesticides, herbicides and industrial additives promotes a less inflammatory and more eubiotic gut environment. Preclinical and observational studies show that chronic exposure to herbicides such as organophosphates and glyphosate induces alterations in the GM, including a reduction in beneficial bacteria, an increase in opportunistic *Proteobacteria*, and a decrease in short-chain fatty acids (SCFAs) production [11,12,13].

In contrast, organic diets have been shown to increase the plasma antioxidant capacity, due to the presence of a high content of polyphenols [11,12]. Organic food significantly reduces exposure to pesticides, contributing to a lower toxic burden on the human body [1,13]. This reduction is particularly relevant given the growing evidence that even short-term dietary exposure to common pesticide classes, such as organophosphates and pyrethroids, can impact GM diversity and metabolic function. In a controlled human study, Ueyama et al. [5] demonstrated that increased urinary levels of pesticide metabolites, such as 3-phenoxybenzoic acid (3-PBA) and dialkylphosphates (DAPs), were correlated with alterations in the abundance of *Akkermansia* and *Bacteroides*, suggesting that GM composition may serve as a sensitive biomarker of environmental chemical intake.

In addition to the well-known metabolic, cardiovascular and inflammatory effects, the Mediterranean Diet plays a key role in modulating the GM. The Mediterranean diet is primarily a plant-based diet. The high intake of soluble fibers, polyphenols, and antioxidant compounds derived from legumes, vegetables, fruits, nuts, extra virgin olive oil and fish promotes increased microbial biodiversity, an abundance of commensal bacteria (e.g., *Faecalibacterium prausnitzii*, *Bifidobacterium* spp., *Akkermansia muciniphila*), and the production of beneficial metabolites such as SCFAs, particularly butyrate and propionate [14,15,16].

The use of organically grown foods within the Mediterranean Diet further reinforces its beneficial effects. A significant reduction in total homocysteine and phosphorus blood values were observed in healthy individuals and in chronic kidney disease patients after 2 weeks of an Italian Mediterranean organic diet (IMOD) [17].

Here, we explore whether dietary interventions in healthy individuals can alter GM communities in a rapid, diet-specific manner. We prepared three intervention diets that varied according to their primary food source and quality: a free diet (No Diet), an IMOD, and an Italian Mediterranean non-Organic Diet (IMnOD). We hypothesize that the adoption of IMOD amplifies favorable impacts on the GM compared to IMnOD and No Diet. Furthermore, we verify the effect of IMOD and IMnOD on anthropometric and body composition parameters.

## 2. Materials and Methods

### 2.1. Study Design

This randomized controlled pilot trial was conducted at the Tor Vergata University of Rome between 1 June and 31 October 2024. An a priori power analysis (paired *t*-test, expected effect size dz = 1, α = 0.05, 80% power) showed that only 10 paired subjects were needed, but to allow for possible drop-outs, we planned to enroll 13 participants per group (39 in total), ensuring roughly 90% power.

A total of 50 unrelated healthy volunteers were initially recruited and screened for eligibility. The inclusion criteria were as follows: (1) adults aged 18–65 years; (2) no history of chronic gastrointestinal diseases (e.g., Crohn’s disease, ulcerative colitis, celiac disease); (3) no antibiotic or probiotic use in the last 3 months; (4) no major dietary changes in the past month; (5) not currently pregnant or breastfeeding; (6) not following any special diet; (7) ability to provide stool samples and complete study questionnaires; (8) willingness to provide informed consent.

Participants underwent comprehensive assessments to gather nutritional and dietary information, including anthropometric and instrumental measurements via bioimpedance analysis (BIA). Specific questionnaires were also administered to evaluate lifestyle and dietary habits, such as the Mediterranean Diet Adherence Screener (MEDAS) questionnaire for Mediterranean Diet adherence and the Food Frequency Questionnaire (FFQ) to analyze food consumption patterns.

The volunteers’ normal bowel frequencies ranged from two times a day to once every other day. Concurrently, fecal samples were collected for analyzing GM composition.

Participants, both males and females, were randomly assigned to one of three study arms: (1) 4 weeks of No Diet; (2) 4 weeks of IMOD; and (3) 4 weeks of IMnOD.

At the start, all participants were following their habitual free unrestricted diet. Participants were then randomly assigned to one of the study arms. The study aimed to compare the effects of IMOD versus IMnOD on the GM and body composition.

### 2.2. Intervention Diets

No Diet: This diet was designed to reflect a free-living dietary pattern representative of each individual’s eating habits. Participants were following their habitual free unrestricted self-selected diet without receiving any nutritional counseling or specific instructions regarding food choices, portion sizes, meal frequency, or dietary composition. No restrictions or monitoring were imposed, and participants were instructed to maintain their usual lifestyle and dietary behavior throughout this period.

IMOD and IMnOD: We constructed two diets, structured following the principles of the Italian Mediterranean Diet [18]. The macronutrient composition of both the IMOD and IMnOD was designed as follows: carbohydrates 50% to 60%, proteins 15% to 20% (with approximately 50% of protein coming from plant-based sources), total fat less than 30% (with saturated fat limited to less than 10% and cholesterol intake restricted to less than 300 mg/day), and 30 g of fibers. Alcohol was restricted, with only 100 mL/day of red wine allowed. The diet was planned to achieve an animal-to-vegetable protein ratio as close to 1:1 as possible. The dietary composition of animal-based foods during the study was as follows: four servings of fish, two servings of meat, and two servings of cheese per week. The only difference between them was the food source: certified organic products for IMOD and conventional products for IMnOD. Organic products for the IMOD group were supplied free of charge by NaturaSì^®^ (Rome, Italy).

Each diet was consumed ad libitum for four consecutive weeks by Italian volunteers between the ages of 18–65. Participants were requested not to change their daily total energy intake (kcal/day), compared with their usual energy intake, during the duration of the study.

Participants’ physical activity levels remained consistent throughout the study period, and no changes in their resting metabolic rate were anticipated.

### 2.3. Data Collection

Patients underwent comprehensive medical, nutritional, and dietary evaluations, which included anthropometric measurements (weight, height, circumferences) and instrumental analysis using BIA. Stool samples were collected to analyze GM composition. Additionally, two questionnaires were administered: the MEDAS [19] was used to evaluate adherence to the Mediterranean diet and the FFQ [20] was used to assess participants’ eating habits.

### 2.4. Anthropometry

The participants’ height and weight were measured using a stadiometer and a scale (Invernizzi, Rome, Italy), with the patients standing and dressed in only their underwear. Height was recorded to the nearest 0.1 cm, while weight was measured to the nearest 0.1 kg [18]. Body mass index (BMI) was calculated by dividing weight (in kilograms) by the square of height (in meters) [21]. According to the World Health Organization (WHO) [22], BMI cut-off points are defined as follows: underweight is classified as a BMI less than 18.5 kg/m^2^, normal weight is classified as 18.5–24.9 kg/m^2^, overweight is classified as 25.0–29.9 kg/m^2^, and obesity is classified as a BMI of 30.0 kg/m^2^ or higher.

Additionally, calf and mid-arm circumferences were measured using a flexible, non-stretchable metric tape as part of the detailed anthropometric evaluations [22]. The waist-to-height ratio (WHR) was determined using a cutoff value of 0.5 [23].

The body adiposity index (BAI) was calculated using the following formula [24]:

BAI = 100 × (hip circumference (m)/(height (m))^1.5^)) − 18

### 2.5. Bioimpedance Analysis (BIA)

Body composition was assessed using BIA with a phase-sensitive device (BIA 101S, Akern/RJL Systems, Florence, Italy) operating at 50 kHz, following standardized procedures [25].

From the BIA analysis, resistance (R) and reactance (Xc) were measured, and several parameters were derived, including fat mass (expressed in both kilograms (FM) and percentage (FM%)), fat-free mass (in kilograms (FFM) and percentage (FFM%)), body cell mass (in kilograms (BCM) and percentage (BCM%)), extracellular mass (ECM), basal metabolic rate (BMR), body cell mass index (BCMI), and phase angle (PhA) [26]. Additionally, total body water was calculated in liters (TBW) and as a percentage (TBW%), using the De Lorenzo equation specifically developed for improved accuracy in obese individuals [27]. The analysis also included extracellular water (in liters (ECW) and percentage (ECW%)) and intracellular water (in liters (ICW) and percentage (ICW%)).

### 2.6. Eating Habits Evaluation

The MEDAS questionnaire comprises 14 items, with 12 addressing the frequency with which specific foods are consumed and 2 focusing on habits associated with the Mediterranean diet. Responses are scored as follows: 1 point for each positive answer and 0 points for each negative answer, resulting in a maximum possible score of 14. A higher score signifies greater adherence to the Mediterranean diet and is associated with a reduced risk of adverse cardiovascular events. In contrast, a lower score indicates poor adherence to the Mediterranean dietary pattern and a corresponding increased risk of health issues. Based on their scores, participants were categorized into three groups: low adherence (0–5 points), medium adherence (6–10 points), and high adherence (above 10 points) [19].

A FFQ was used to evaluate their weekly consumption patterns for various foods. The FFQ measured the frequency of intake for 36 foods commonly consumed in Italy, as well as their portion sizes. Compliance rates were calculated for each food item [20].

### 2.7. Gut Microbiota

All participants provided two stool samples: one collected the day before the start of the assigned dietary protocol (baseline), and the other collected in the morning following the final day of the 4-week intervention period. Each patient received a standardized collection kit accompanied by detailed written and verbal instructions to ensure the proper handling and collection of fecal samples. These guidelines were designed to minimize the risk of contamination and preserve sample integrity for downstream analysis. Stool samples were collected and processed for 16S rRNA gene sequencing. GM analysis was conducted by Wellmicro^®^ (Via Antonio Canova, 30, 40138 Bologna BO, Italy) [28].

### 2.8. Statistical Analysis

Data collection was realized through Microsoft Office Excel^®^ (2020, Microsoft, Redmond, WA, USA). Descriptive statistics were conducted for the demographic and anthropometric characteristics of patients. Continuous variables were summarized using the mean and standard deviation (SD), while categorical variables were presented as frequency and percentage.

The Shapiro–Wilk test was performed to assess the normality of the sample distribution. Based on this, we employed either an unpaired *t*-test or a nonparametric Wilcoxon test to assess differences in the nutritional parameters between No diet, IMOD and IMnOD. An approximate one-way analysis of variance (ANOVA) was performed to compare the means of three groups (No Diet, IMOD, and IMnOD) across anthropometric characteristics and bioimpedance results.

#### 2.8.1. Taxonomic Profiling and Multivariate Analyses

Differentially abundant microbial taxa across dietary phases were identified using sparse Partial Least Squares Discriminant Analysis (sPLS-DA), implemented in the mixOmics R package (R version 4.3.2 (31 October 2023)) [29]. Counts of Operational Taxonomic Units (OTUs) were extracted, log-transformed using the natural logarithm plus one (log1p), and scaled. The model included two components, selecting the top 50 discriminative taxa per component (keepX = c (50, 50)).

To compare differences among the three groups, the structure of the microbial community was assessed using the following: (a) Alpha Diversity: calculated via the Shannon index, and (b) Beta Diversity: with Bray–Curtis dissimilarity, visualized with Principal Coordinates Analysis (PCoA) plots and statistically evaluated using Permutational Multivariate Analysis of Variance (PERMANOVA).

Associations between the dietary intervention and alpha diversity were assessed using linear mixed-effects models fitted by Restricted Maximum Likelihood (REML). The models accounted for repeated measures by including the diet group and fixed effect and the subject ID as a random effect. Statistical significance was evaluated using Satterthwaite’s approximation for degrees of freedom to compute *p*-values. For beta diversity, differences in the composition of the microbial community between diet groups were assessed using PERMANOVA based on Bray–Curtis dissimilarity matrices.

Pearson correlation analyses were performed to investigate the associations between microbial taxa and anthropometric, dietary, and bioimpedance variables. Correlation matrices were visualized for each dietary phase, highlighting significant associations (*p* < 0.05).

#### 2.8.2. Sex-Based Microbiota Variations

To further investigate potential sex-based differences in the microbiota’s response to dietary intervention, a stratified analysis was performed. A PERMANOVA test considering both the intervention type and biological sex and Dispersion analysis (beta-dispersion) was used to assess the within-group variability, stratified by sex across intervention deltas (ΔIMOD, ΔIMnOD, ΔNoDiet). Sex-specific sPLS-DA analyses were conducted to identify taxa that most contributed to separating the intervention groups within each sex. Comparative loading plots were considered in order to reveal distinct microbial patterns between sexes.

All statistical analyses were performed using R (version 4.4.2). *p* < 0.05 was considered statistically significant.

## 3. Results

### 3.1. Study Participants and Baseline Characteristics

A total of 50 participants were initially enrolled in the study based on the inclusion criteria. Following the baseline assessment, nine participants withdrew due to their unwillingness to continue with the follow-up schedule. Two additional participants were excluded during the study: one due to pregnancy and the other after testing positive for COVID-19.

Therefore, 39 participants (27 women 69.23% and 12 men 30.77%) with a mean age of 38.46 ± 10.35 years completed the study. The general characteristics of the study population are reported in Table 1.

### 3.2. Nutritional Composition of the Dietary Interventions

In order to assess any differences between the IMOD and IMnOD, a nonparametric Wilcoxon test was conducted. The nutritional characteristics did not reveal any statistically significant differences, as reported in Table 2.

All groups were balanced for sex (nine females and four males per group), with no statistically significant differences in age, baseline anthropometric characteristics and body composition. An ANOVA test was conducted and is reported in Table 3.

Similarly, BIA parameters did not reveal any significant differences between the groups, as reported in Table 4.

The MEDAS score for each dietary phase reported no significant differences between the IMOD and IMnOD (IMOD: 9.69 ± 1.93; IMnOD: 10.08 ± 2.28; *p* = 0.51).

### 3.3. Gut Microbiota Diversity and Composition Across Dietary Interventions

**Figure 1:**
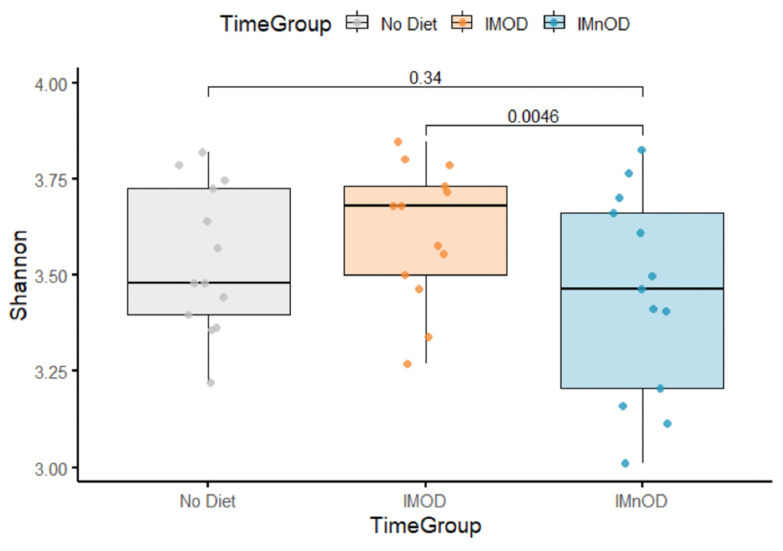
Boxplot of Shannon diversity values across dietary groups: No diet, IMOD, and IMnOD. Boxes represent the interquartile range, horizontal lines indicate medians, and whiskers denote minimum and maximum values. Abbreviations: IMnOD, Italian Mediterranean non-Organic Diet; IMOD, Italian Mediterranean Organic Diet; No Diet: free diet.

The aggregated data regarding the alpha diversity in the three dietary groups (No Diet, IMOD and IMnOD), assessed using the Shannon index, are reported in Figure 1. The median Shannon diversity showed a consistent trend of being higher in the IMOD group compared to the IMnOD group.

Pairwise *p*-values were initially computed using paired Wilcoxon tests to compare the alpha diversity (Shannon index) between dietary groups within the same subjects over time, and a statistical significant difference was reported between the IMOD and IMnOD. To account for both fixed effects (diet) and random effects (subject-specific variation), we subsequently applied a linear mixed-effects model fitted by restricted maximum likelihood (REML). The results of the linear mixed-effects model fitted by REML indicated that the IMOD was associated with a statistically significant difference in the alpha diversity compared to the IMnOD (Δ = 0.16; *p* = 0.01). In contrast, no statistically significant difference was observed between the IMOD and the No Diet group (Δ = 0.09; *p* = 0.14).

**Figure 2:**
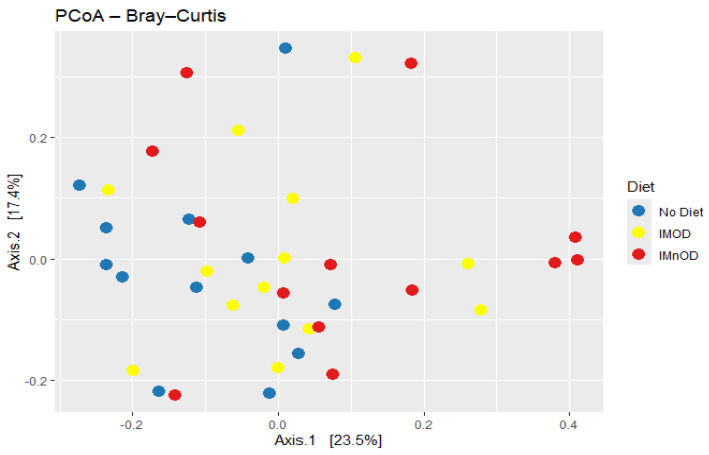
Beta diversity analysis using Bray–Curtis dissimilarity. PCoA plot showing sample distribution based on Bray–Curtis dissimilarity across different diet groups: No diet, IMOD and IMnOD. Abbreviations: IMnOD, Italian Mediterranean non-Organic Diet; IMOD, Italian Mediterranean Organic Diet; No Diet: free diet.

**Figure 3:**
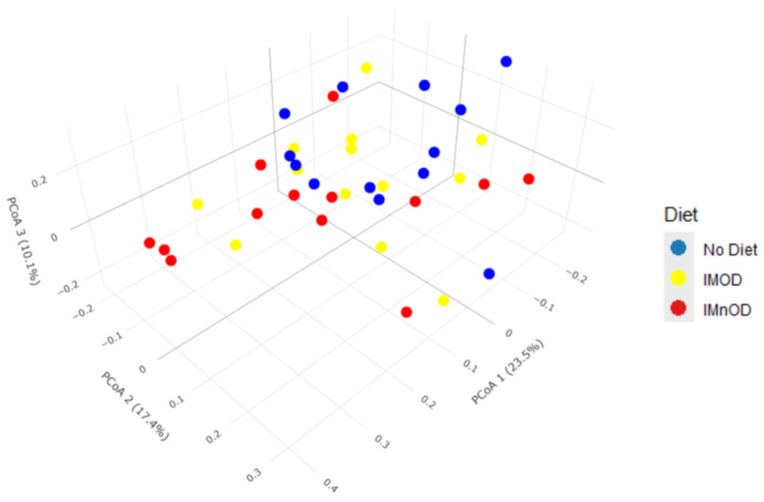
Three-dimensional PCoA plot based on Bray–Curtis dissimilarity, visualizing the separation of gut microbiota profiles across the No diet, IMOD, and IMnOD groups. Each point represents a participant’s sample colored by diet group. Abbreviations: IMnOD, Italian Mediterranean non-Organic Diet; IMOD, Italian Mediterranean Organic Diet; No Diet: free diet.

Figure 2 and Figure 3 display the distribution of samples along the first three principal coordinates derived from Bray–Curtis dissimilarity, which together capture a substantial portion of the variation in gut microbial composition (Axis 1 = 23.5%, Axis 2 = 17.4%, Axis 3 = 10.4%). As shown in the 2D and 3D PCoA plots, samples from the IMnOD group (red) tend to cluster toward the right side of Axis 1, partially separated from the No Diet group (blue), which is more dispersed and concentrated in the left quadrant. The IMOD group (yellow) exhibits an intermediate distribution, overlapping both other groups, but with several individuals extending along Axis 2.

This spatial separation is supported by the PERMANOVA analysis (R^2^ = 0.0736, *p* = 0.001), indicating that dietary intervention explains approximately 7.4% of the total variance in microbial community composition.

Moreover, a PERMANOVA based on intra-subject microbial shifts (Δ) was performed. It showed significant differences in the composition of the microbiota across interventions (*p* = 0.048, R^2^ = 0.077), despite the moderate effect size.

**Figure 4:**
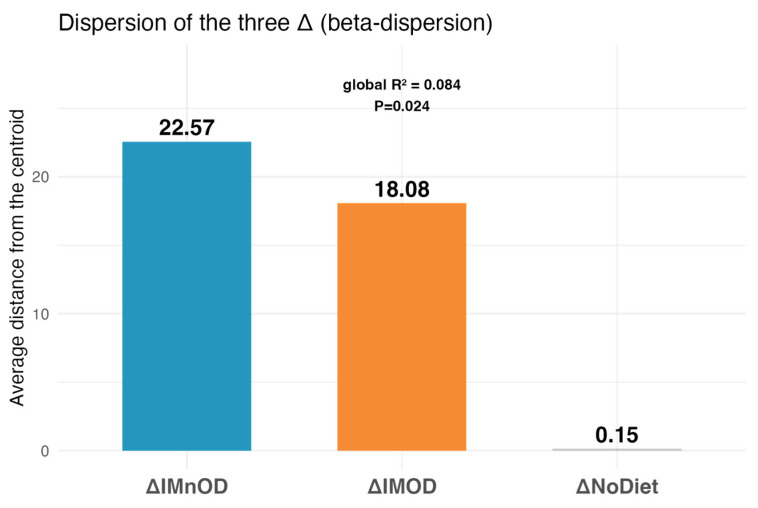
Within-group beta-dispersion across dietary interventions. Average Euclidean distance to group centroid for each intervention arm (ΔIMnOD, ΔIMOD, ΔNoDiet). These distances reflect the degree of intra-individual microbial shifts between baseline and post-intervention timepoints, referred to as delta (Δ) changes. Specifically, ΔIMOD indicates the change over time in the Italian Mediterranean Organic Diet group, ΔIMnOD refers to changes in the Italian Mediterranean non-Organic Diet group, and ΔNoDiet represents the change observed in the free diet (control) group.

Additionally, beta-dispersion analysis revealed significant differences in the within-group variability (*p* = 0.024, R^2^ = 0.084), with the highest dispersion in the IMnOD, moderate dispersion in the IMOD, and minimal dispersion for No Diet (Figure 4).

### 3.4. sPLS-DA and Microbial Discriminant Signatures

**Figure 5:**
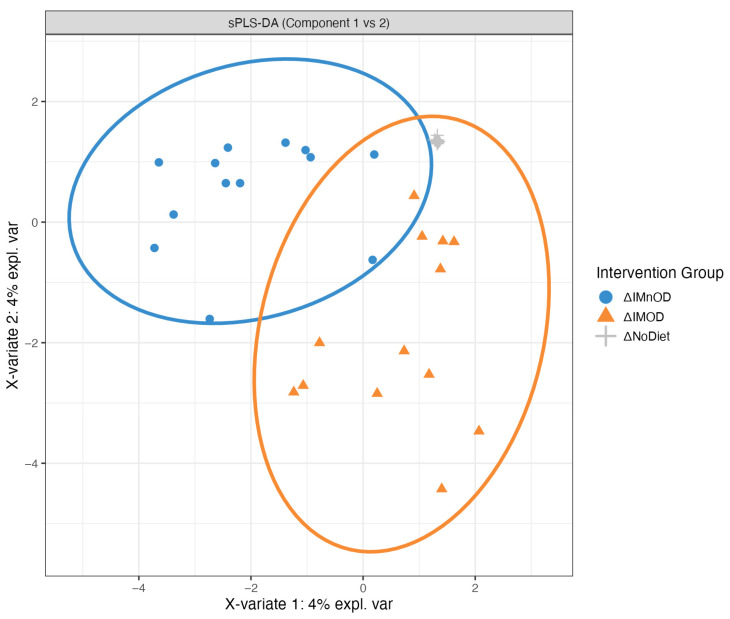
sPLS-DA plot showing microbial separation across dietary interventions. Discriminant analysis of microbial community shifts using sparse Partial Least Squares Discriminant Analysis (sPLS-DA), based on delta changes (Δ) in microbiota composition. Distinct clustering is observed between the IMnOD (blue circles) and IMOD (orange triangles) groups, with minimal overlap. The No Diet group (gray crosses) shows little separation.

To identify microbial signatures associated with dietary interventions, we applied sparse Partial Least Squares Discriminant Analysis (sPLS-DA) to delta changes (Δ) data. A clear separation between the ΔIMOD and ΔIMnOD groups was observed, with minimal overlap and tighter clustering in the IMOD group. Distinct microbiota trajectories induced by the two diets were reported (Figure 5).

**Figure A1:**
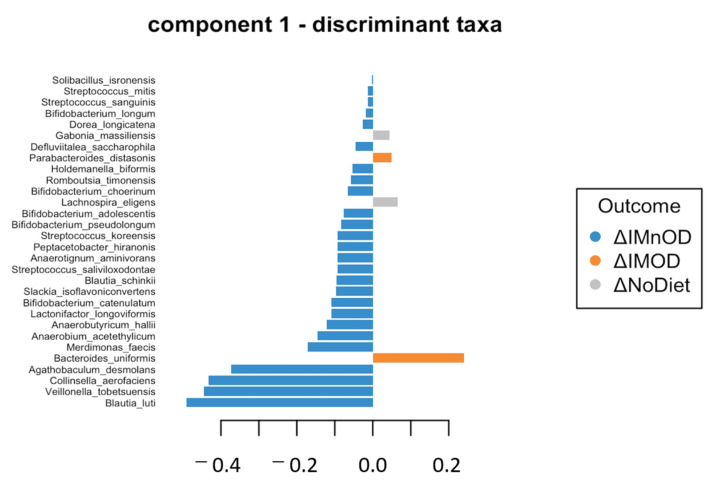

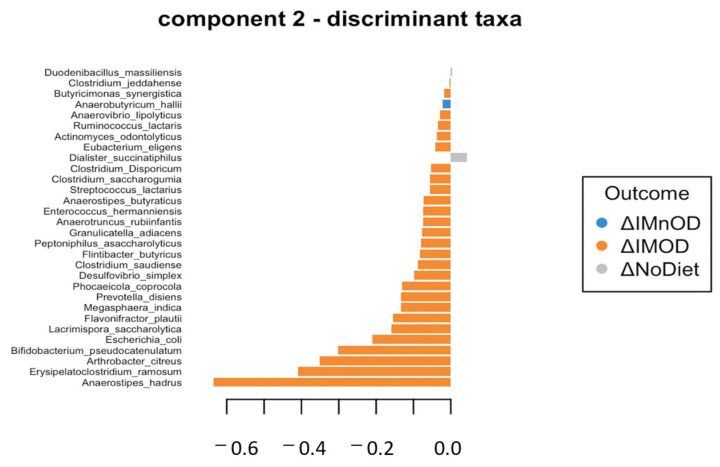
Discriminant taxa associated with Component 1 and Component 2 obtained from sPLS-DA analysis to Δ data. Bar plot showing the top taxa contributing to separation among dietary groups along Component 1 and 2 of the sparse Partial Least Squares Discriminant Analysis (sPLS-DA). Taxa are color-coded according to their primary association: orange for IMOD (Italian Mediterranean Organic Diet), blue for IMnOD (non-Organic), and grey for No Diet.

The discriminant taxa associated with Component 1 and Component 2 are shown in Figure A1 (Appendix A). Component 1 primarily separated ΔIMnOD participants, with top contributors including *Blautia luti*, *Veillonella tobetsuensis*, *Collinsella aerofaciens*, and *Agathobaculum desmolans*. Conversely, taxa such as *Bacteroides uniformis* were associated with the IMOD. Component 2 revealed a microbial profile strongly enriched in the IMOD group, including *Anaerostipes hadrus*, *Erysipelatoclostridium ramosum*, *Arthrobacter citreus*, *Escherichia coli*, and *Bifidobacterium pseudocatenulatum.*

The top 30 discriminative microbial features obtained by the PLS-DA of the three groups are reported in Table 5.

### 3.5. Sex-Specific Effects on Gut Microbiota Composition

**Figure 6:**
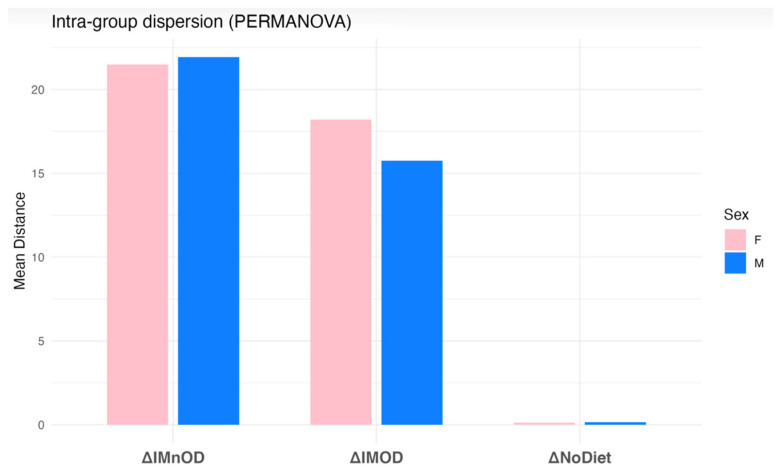
Two-way PERMANOVA including diet and sex revealed that both factors significantly influence GM composition (*p* = 0.014).

A two-way PERMANOVA analysis including both diet and sex factors revealed that both variables significantly influenced GM composition (*p* = 0.014, R^2^ = 0.1215), (Figure 6).

**Figure A2:**
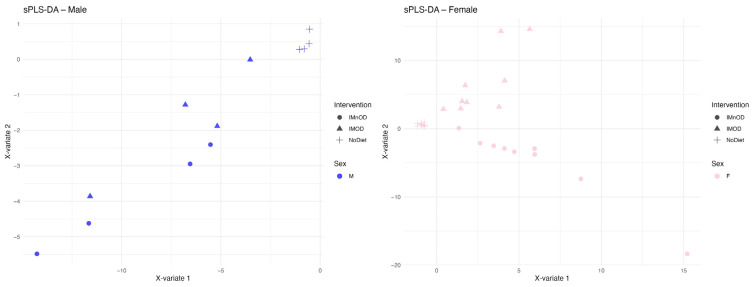
Sex-stratified beta-dispersion analysis showing average intra-group distances (Euclidean) from individual samples to group centroids for each intervention group. ΔIMnOD refers to the change in microbiota composition over time in the Italian Mediterranean non-Organic Diet group, ΔIMOD refers to the change in the Italian Mediterranean Organic Diet group, and Δ NoDiet refers to changes in the free diet control group. Pink bars represent females and blue bars represent males. Greater distances indicate higher intra-group variability.

To further explore intra-group variability, beta-dispersion analysis was performed based on the Euclidean distances to group centroids. As shown in Figure A2 (Appendix A), dispersion patterns varied across intervention groups and sexes.

**Figure 7:**
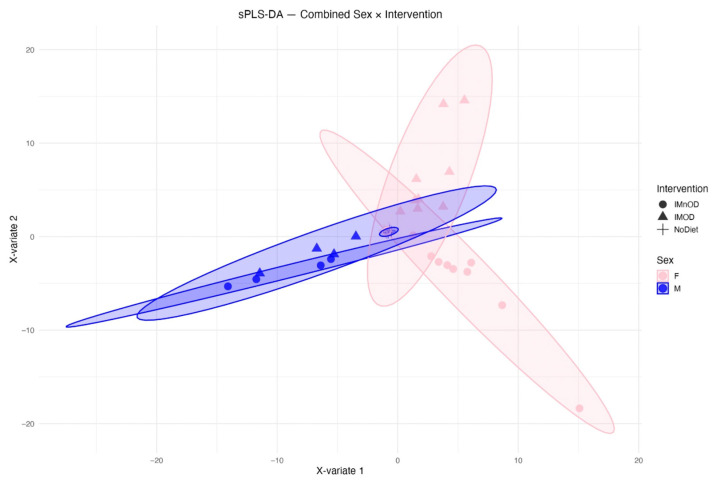
sPLS-DA (Sparse Partial Least Squares Discriminant Analysis) plots showing composition of the gut microbiota stratified by sex and intervention. Each point represents an individual, positioned according to the first two sPLS components (X-variate 1 and 2). Shapes indicate dietary intervention, while colors indicate sex (pink for females, blue for males).

Figure 7 further illustrates these sex-specific responses using sPLS-DA plots stratified by sex. Among females, a clear separation between intervention groups (IMOD, IMnOD, NoDiet) was observed, suggesting that dietary intervention produced distinguishable shifts in the composition of the gut microbiota. In contrast, the sPLS-DA for males showed more compact clustering and less separation between groups, possibly due to the limited sample size (*n* = 4 per group).

**Figure 8:**
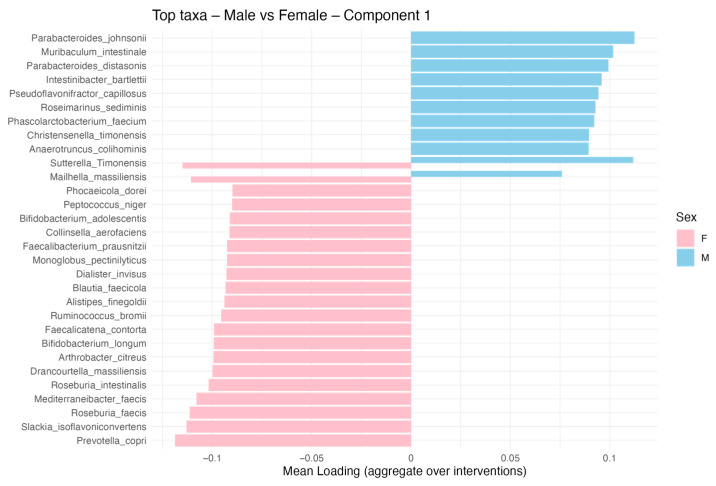

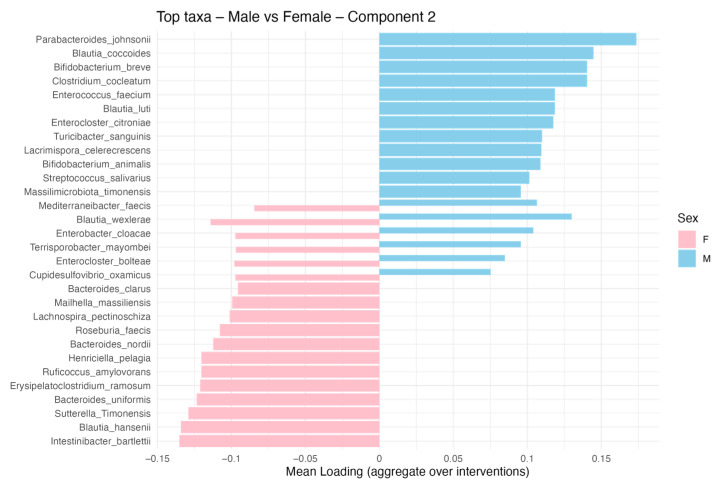
Discriminant microbial taxa by sex based on sPLS-DA Component 1 and 2. Bar plot showing the top microbial taxa contributing to the separation between male (blue) and female (pink) participants. Taxa were identified using sparse Partial Least Squares Discriminant Analysis (sPLS-DA) applied to the composition of the gut microbiota.

Moreover, distinct microbial signatures were identified for each sex: in men (blue), taxa such as *Parabacteroides johnsonii*, *Muribaculum intestinale*, *Parabacteroides distasonis* and *Intestinibacter barletti* displayed a high discriminative power for Component 1. Along Component 2, *Parabacterioides johnsonii*, *Blautia coccoides*, *Bifidobacterium breve*, *Clostridium cocleatum* and *Enterococcus faecium* were also dominant. In women (pink), the GM was characterized by the enrichment of *Prevotella copri*, *Roseburia faecis*, *Sklackia isofalvoniconvertens Mediterraneaneibacter faecis*, *Faecalibacterium prausnitzii*, *Collinsella aerofaciens* and *Sutterella timonensis* for Component 1. On Component 2, taxa such as *Sutterella timonensis*, *Intestinibacter bartlettii*, *Blutia hansenii* and *Bacteroides uniforms* were more strongly represented. Components 1 and 2 are reported in Figure 8.

**Figure A3:**
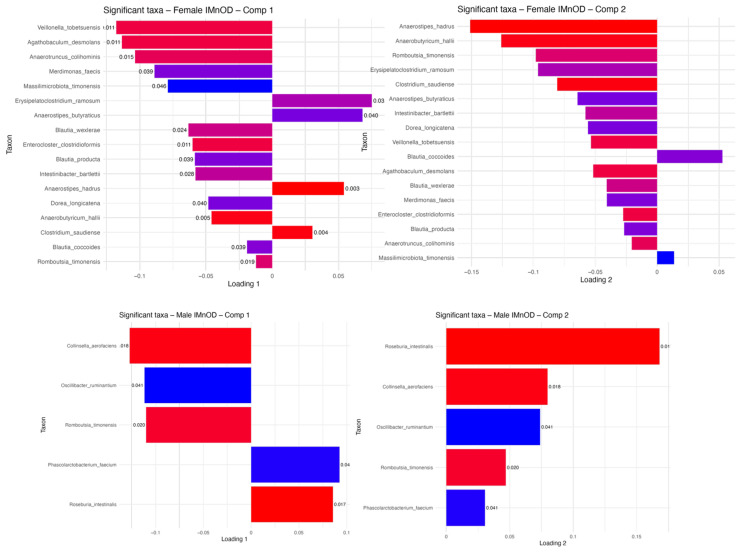
Sex-specific discriminant microbial taxa in the IMnoD group based on sPLS-DA.

**Figure A4:**
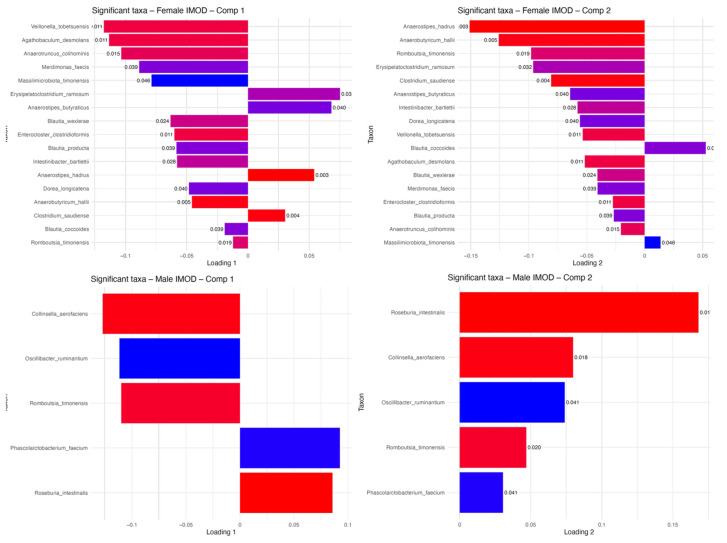
Sex-specific discriminant microbial taxa in the IMOD group based on sPLS-DA.

**Figure A5:**
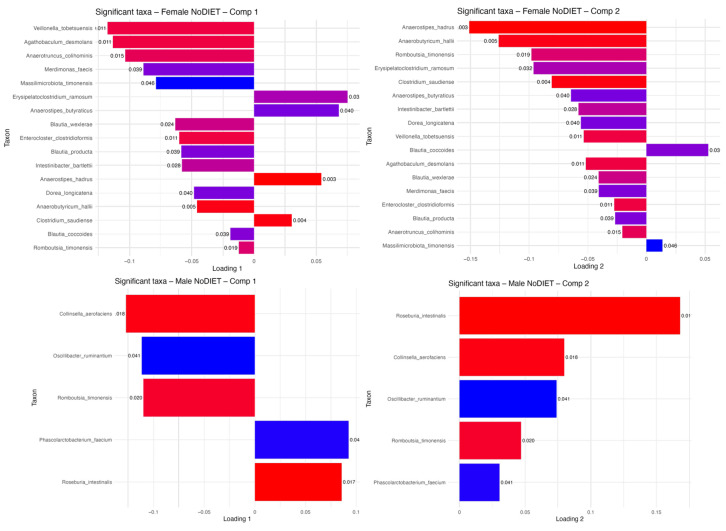
Sex-specific discriminant microbial taxa in the No Diet group based on sPLS-DA.

The sPLS-DA revealed sex-specific microbial signatures within the group, across Component 1 and Component 2, indicating distinct taxonomic drivers of variability for males and females, as reported in Figure A2, Figure A3, Figure A4 and Figure A5 (Appendix A).

### 3.6. Correlation Analysis Between Microbiota and Body Composition Parameters

**Figure 9:**
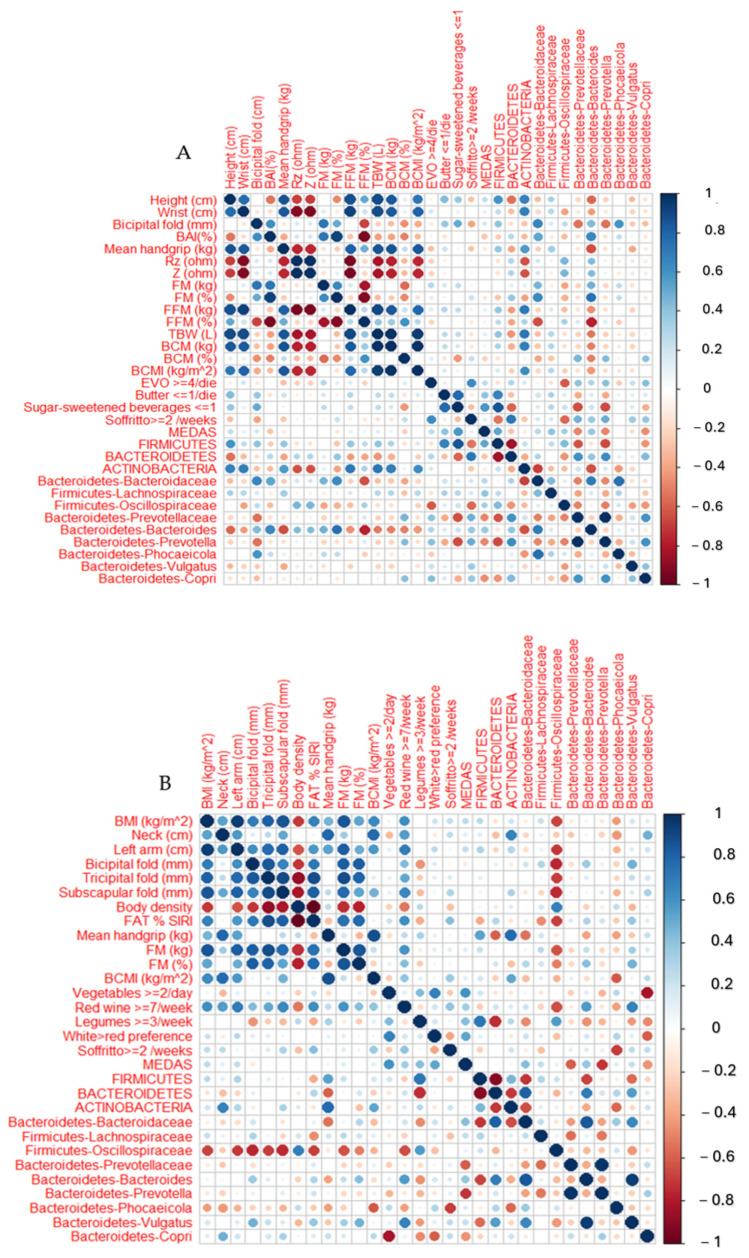

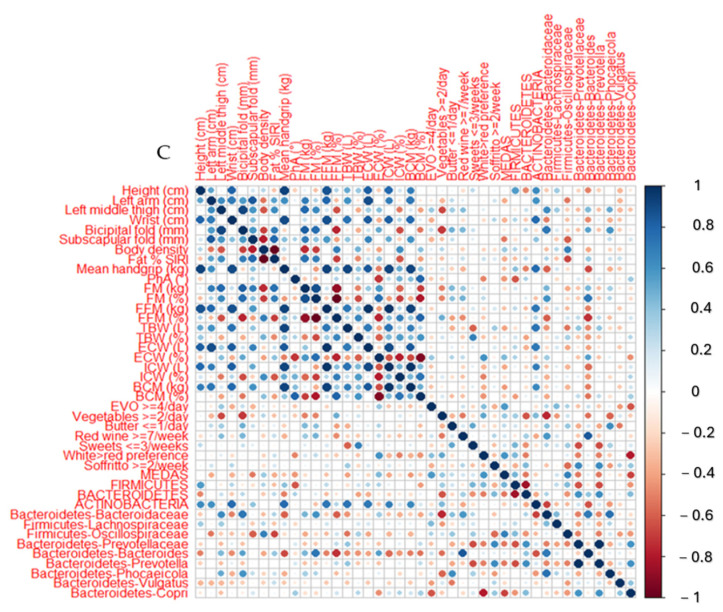
Correlation matrices of Spearman correlation coefficients between gut microbiota and clinical, anthropometric, and dietary variables for each dietary phase: (**A**) No diet, (**B**) IMOD, and (**C**) IMnOD groups. Circle size and color intensity indicate the strength and direction of correlations (blue = positive, red = negative). Only statistically significant correlations (*p* < 0.05) are reported. Abbreviations: BAI, body adiposity index; BCM, Body Cell Mass; BCMI, Body Cell Mass Index; BMI, body mass index; ECW, Extracellular Water; ICW, Intracellular Water; PhA, Angle Phase; RZ, Resistance; TBW, Total Body Water; WHR, waist hip ratio; Xc, Reactance; Z, Impedance.

A correlation matrix was computed to explore the associations between GM and a wide range of anthropometric measures, bioimpedance parameters, and dietary habits. Figure 9 reports several significant correlations.

**Figure A6:**
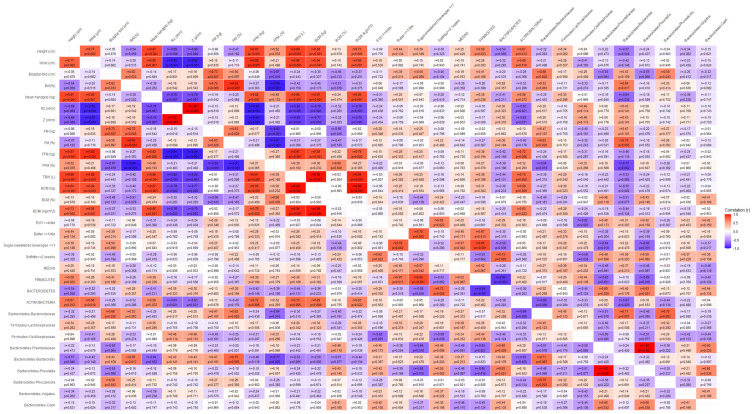
This heatmap illustrates Pearson correlation coefficients (r) between anthropometric measures, dietary variables, and microbial taxa at the phylum and genus levels in the No diet Group. Each cell displays the correlation value (r) along with the corresponding *p*-value, indicating the statistical significance of the associate. Red shades represent positive correlations (r > 0), while blue shades denote negative correlations (r < 0). The intensity of color reflects the strength of the correlation (from −1.0 to +1.0). *p*-values are explicitly shown.

The genus *Bacteroidetes Bacteroides* was positively associated with BAI (*r* = 0.64, *p* = 0.02) and inversely correlated with mean handgrip strength (*r* = −0.64, *p* = 0.03). In contrast, the phylum *Actinobacteria* showed significant positive correlations with parameters indicative of lean mass, including FFM % (*r* = 0.71, *p* = 0.005), BCM (kg) (*r* = 0.70, *p* = 0.009), and BCMI (*r* = 0.62, *p* = 0.02) (Figure 9A and Figure A6).

**Figure A7:**
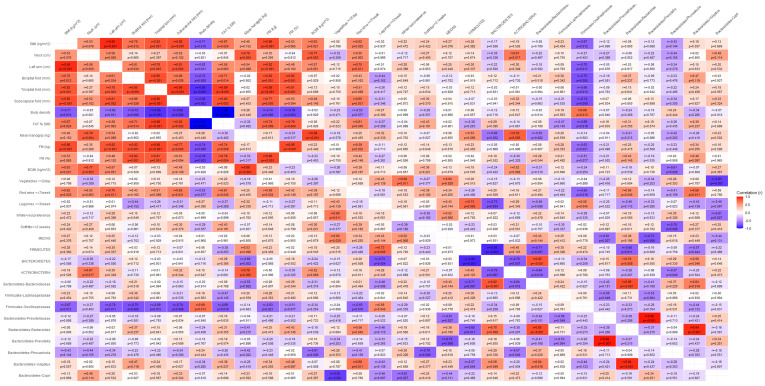
This heatmap illustrates the Pearson correlation coefficients (r) between anthropometric measures, dietary variables, and microbial taxa at the phylum and genus levels in the IMOD group. Each cell displays the correlation value (r) along with the corresponding *p*-value, indicating the statistical significance of the associate. Red shades represent positive correlations (r > 0), while blue shades denote negative correlations (r < 0). The intensity of color reflects the strength of the correlation (from –1.0 to +1.0). *p*-values are explicitly shown.

In the IMOD group (Figure 9B and Figure A7), the family *Firmicutes Oscillospiraceae* was significantly and inversely correlated with several anthropometric and body composition parameters, including BMI (*r* = −0.68, *p* = 0.01), left arm circumference (*r* = −0.70, *p* = 0.01), bicipital skinfold (*r* = −0.75, *p* = 0.004), tricipital skinfold (*r* = −0.70, *p* = 0.01), subscapular skinfold (*r* = −0.74, *p* = 0.008), and FM (kg) (*r* = −0.63, *p* = 0.02). Furthermore, *Oscillospiraceae* abundance was significantly associated with dietary habits, showing an inverse correlation with red wine consumption per week (*r* = −0.65, *p* = 0.01) and a positive correlation with legume intake more than three times a week (*r* = 0.57, *p* = 0.04). Additionally, *Actinobacteria* were positively correlated with mean handgrip strength (*r* = 0.78, *p* = 0.007). The phylum *Firmicutes* showed a strong positive correlation with legume consumption (*r* = 0.72, *p* = 0.004), whereas the *Bacteroides* abundance was inversely correlated with legume intake (*r* = 0.73, *p* = 0.004) and positively correlated with red wine consumption (*r* = 0.56, *p* = 0.04). Lastly, *Bacteroidetes Copri* showed a significant negative correlation with vegetable intake (*r* = −0.82, *p* = 0.0005).

**Figure A8:**
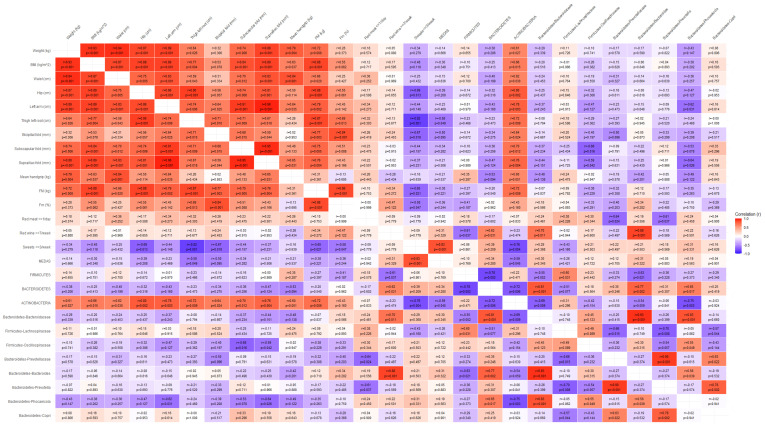
This heatmap illustrates the Pearson correlation coefficients (r) between anthropometric measures, dietary variables, and microbial taxa at the phylum and genus levels in the IMnOD Group. Each cell displays the correlation value (r) along with the corresponding *p*-value, indicating the statistical significance of the associate. Red shades represent positive correlations (r > 0), while blue shades denote negative correlations (r < 0). The intensity of color reflects the strength of the correlation (from −1.0 to +1.0). *p*-values are explicitly shown.

In the IMnOD group (Figure 9C and Figure A8), the *Actinobacteria* abundance was negatively correlated with BMI (*r* = −0.74, *p* = 0.006), waist circumference (*r* = −0.62, *p* = 0.03), hip circumference (*r* = −0.80, *p* = 0.001), left arm circumference (*r* = −0.68, *p* = 0.002), bicipital skinfold (*r* = −0.75, *p* = 0.02), subscapular skinfold (*r* = −0.69, *p* = 0.01), and FM (kg) (*r* = −0.71, *p* = 0.008). Dietary correlations revealed that a higher Bacteroides abundance was associated with red wine consumption (*r* = 0.62, *p* = 0.03); specifically, *Bacteroidete Bacteroides* showed a strong positive association (*r* = 0.80, *p* = 0.0001). Additionally, *Bacteroidetes Prevotellaceae* and *Bacteroidetes Prevotella* were positively associated with meat consumption (*r* = 0.64, *p* = 0.02 and *r* = 0.60, *p* = 0.03, respectively).

## 4. Discussion

This randomized controlled pilot trial demonstrated that adherence to the Mediterranean Diet, whether based on organic or conventional food products, significantly modulates the composition of the GM compared to No diet. Anthropometric and bioimpedance measures did not differ significantly across dietary groups, suggesting that the primary impact of the intervention occurred at the microbial and functional level, rather than in short-term body composition. Compared to the No Diet group, both the IMOD and IMnOD interventions led to an increased relative abundance of several taxa previously associated with SCFA production, including *Anaerobutyricum hallii*, *Anaerostipes hadrus*, and *Dorea longicatena*. These microbial shifts may reflect the influence of the Mediterranean dietary pattern on gut microbial ecology. The gut microbiota encompasses a wide array of bacterial taxa with distinct metabolic activities that can affect the host’s physiology through complex immunometabolic interactions [30].

Importantly, while both dietary interventions resulted in compositional shifts distinct from those observed in the No Diet group, the data did not support a globally greater impact of IMOD compared to IMnOD in terms of overall microbial modulation. Nonetheless, *Parabacteroides distasonis* was uniquely increased in the IMOD and reduced in the IMnOD, suggesting a possible diet-specific microbial adaptation. Moreover, *Anaerostipes hadrus* showed a comparatively greater increase in the IMOD. Both taxa are recognized SCFA producers, notably of butyrate and acetate, and have been implicated in mechanisms related to intestinal barrier maintenance and metabolic regulation [31,32]. Notably, *Parabacteroides distasonis* is known to metabolize polysaccharides into succinate and acetate, and has been linked to the modulation of bile acids and metabolic regulation [33], while *Anaerostipes hadrus* contributes to butyrate synthesis from lactate [34].

Our comparative analysis revealed that *Veillonella tobetsuensis* was elevated in the IMnOD group, a finding consistent with the known capability of *Veillonella* spp. to ferment lactate into acetate and propionate, SCFAs linked to gut homeostasis [35]. Indeed, SCFAs serve as energy sources for colonocytes, regulate immune tolerance by promoting regulatory T cell differentiation, strengthen gut barrier function, and contribute to lipid and glucose metabolism [31,36,37].

Moreover, we found that *Collinsella aerofaciens* and *Agathobaculum desmolans* increased in both the IMOD and IMnOD but reached higher levels in the IMnOD. *Collinsella* has been associated with carbohydrate fermentation and cholesterol metabolism [38], while *Agathobaculum* [39], although less studied, is related to SCFA production. *Anaerobutyricum hallii*, a well-characterized butyrate and propionate producer [40], showed a strong increase in IMOD with a moderate rise in IMnOD. This bacterium’s role in metabolizing lactate and acetate into beneficial SCFAs underpins its recognized association with metabolic health [40]. *Dorea longicatena* followed a similar significative trend; even though it is not a major SCFA producer, some studies suggest that it may influence gut barrier function and host metabolism [31]. *Clostridium saudiense* was also elevated in both diets, particularly the IMOD, and reduced in No Diet, consistent with the genus *Clostridium*’s role in fermentative SCFA pathways [41]. Collectively, these observations show that both Mediterranean diets enrich a range of SCFA-associated taxa, with some differences between organic and conventional sources. While our data do not directly measure metabolic outputs, the enrichment of known SCFA-generating bacteria could support the hypothesis that these dietary interventions may enhance SCFA production, though functional assays would be needed to confirm this in our study context. These results may be attributed not only to the nutrient-dense profile of the Mediterranean dietary pattern but also to the intrinsic quality of organic food products. Organic agriculture is a production system grounded in ecological principles that promotes soil and ecosystem health, biodiversity, and sustainable resource use, while avoiding synthetic pesticides, herbicides, and fertilizers [42]. This reduced chemical burden may partly explain the superior impact of the IMOD on GM composition, as a lower intake of xenobiotics such as pesticide residues and synthetic additives can influence microbial diversity and functionality. Indeed, this reduced exposure contributes to a more favorable exposome, which in turn affects host–microbe interactions [1]. Specifically, pesticides have been shown to alter microbial diversity and favor taxa associated with pro-inflammatory states, whereas organic food consumption supports the colonization of SCFA-producing bacteria and the preservation of mucosal immunity. These immunometabolic effects may reflect the key mechanism through which IMOD promotes gut and systemic health, especially via enhanced SCFA biosynthesis, improved epithelial barrier integrity, and the modulation of Treg cell function [43]. Furthermore, recent studies demonstrate that a lower pesticide burden is associated with higher microbial diversity and functional potential, including enhanced SCFA biosynthesis pathways [44].

Moreover, evidence suggests that organically grown foods contain 10–50% higher concentrations of bioactive phytochemicals, including polyphenols and antioxidants, compared to conventionally grown products [45]. The integration of organic cultivation within the Mediterranean Diet framework may further potentiate its health benefits by enhancing the intake of phytochemicals that act as key modulators of the gut microbiota and systemic inflammation. Organic farming practices, which avoid synthetic pesticides and prioritize soil biodiversity, are aligned with the ecological and cultural foundations of the traditional Mediterranean food system. This synergy not only improves the nutritional density of foods but also supports a sustainable dietary model that promotes both human and environmental health. In this context, the IMOD represents a coherent evolution of the traditional Mediterranean pattern, preserving its core principles while minimizing exposure to environmental contaminants and maximizing the intake of health-promoting compounds such as polyphenols, flavonoids, and antioxidants [45]. These compounds are known to influence microbial composition and intestinal barrier function, potentially explaining the higher abundance of beneficial taxa observed following the IMOD. Additional evidence supports the role of the IMOD in modulating GM and reducing inflammatory and cardiometabolic risk [8,46]. For instance, in a recent trial, individuals adhering to an IMOD exhibited improvements in insulin sensitivity, lipid profile, and microbial alpha-diversity, independent of energy intake or weight changes [8]. Moreover, Jiang et al. demonstrated that regular organic food consumers present lower markers of oxidative stress and systemic inflammation, possibly due to an enhanced antioxidant and polyphenol intake, which in turn shapes the GM towards more eubiotic configurations [45].

In light of our findings, it is important to consider the potential influence of individual characteristics, such as sex, on GM dynamics. A PERMANOVA analysis including both the dietary intervention (expressed as delta changes) and sex as explanatory variables revealed a statistically significant impact on GM composition (R^2^ = 0.1215, F = 1.6135, *p* = 0.014), indicating that these two factors jointly explain 12.15% of the variance in microbial community structure. Notably, the IMnOD group exhibited the highest intra-group variability across both sexes, suggesting a more heterogeneous microbial response to this intervention. In contrast, IMOD elicited more consistent microbial modulation, as reflected by the lower within-group variability. Interestingly, the No Diet condition showed relatively stable GM profiles, especially among males, potentially reflecting the absence of dietary perturbation.

In support of these findings, the sPLS-DA revealed sex-specific taxa contributing to microbial differentiation. Notably, in men, *Parabacteroides distasonis*, a bacterium capable of fermenting polysaccharides into succinate and acetate and modulating bile acid metabolism, has been shown to improve metabolic health and reduce hepatic fibrosis in preclinical models [47]. *Muribaculum intestinale* remains less characterized but is implicated in carbohydrate fermentation in murine gut ecosystems, and *Parabacteroides johnsonii* has been reported to stabilize gut microbial diversity in murine studies [48].

In women, *Prevotella copri* was prominent. This species has been variably linked to carbohydrate metabolism and immune modulation, including associations with rheumatoid arthritis via the promotion of Th17-driven inflammatory responses [48]. *Roseburia faecis*, a well-established butyrate producer, contributes to mucosal integrity and resilience, with demonstrated protective effects in stress-evoked murine models [49]. *Collinsella aerofaciens* plays a role in carbohydrate metabolism and cholesterol handling, and may exert estrogen-relevant immunometabolic effects, suggestive of involvement in the estrobolome [50]. Lastly, *Sutterella timonensis*, known for its mild pro-inflammatory activity and potential to degrade IgA, has been observed in association with altered mucosal immunity in inflammatory conditions [51].

Furthermore, *Faecalibacterium prausnitzii* emerged as the microbial taxon most strongly associated with the female component, exhibiting a higher mean loading value in driving the separation of GM composition. Among the most relevant SCFA-producing taxa, *Faecalibacterium prausnitzii* plays a pivotal role in maintaining gut homeostasis. As one of the most abundant commensals in the healthy colon, *F. prausnitzii* exerts anti-inflammatory effects through the production of butyrate and unique bioactive molecules such as the Microbial Anti-inflammatory Molecule (MAM), which modulates NF-κB signaling and preserves epithelial integrity. Recent reviews have underscored its central role in the gut ecosystem, highlighting not only its protective action in inflammatory bowel diseases but also its capacity to shape broader microbial networks by producing cross-feeding substrates that sustain other beneficial microbes [52].

Moreover, new evidence suggests that *F. prausnitzii* contributes to host metabolic regulation beyond SCFA production. Specifically, it expresses a fatty acid amide hydrolase (FAAH) enzyme capable of degrading bioactive N-acyl amides such as oleoylethanolamide (OEA) and palmitoylethanolamide (PEA), which are implicated in satiety signaling, gut motility, and immune modulation. This enzymatic function represents a novel interface between microbial metabolism and host endocannabinoid pathways, offering mechanistic insight into the potential systemic benefits of diets that enhance *F. prausnitzii* abundance [53]. These findings underscore the need to consider sex-specific microbial signatures when evaluating diet–microbiota–host interactions, while cautioning that our study did not directly assess functional outputs or host biomarkers

Moreover, to our knowledge, for the first time, our correlation analyses consistently reveal that *Actinobacteria* are positively associated with FFM% (r = 0.71, *p* = 0.005), BCM (r = 0.70, *p* = 0.009), and BCMI (r = 0.62, *p* = 0.02) in the No Diet group, as well as with the mean handgrip strength (r = 0.78, *p* = 0.007) in the IMOD group. Conversely, in the IMnOD group, *Actinobacteria* abundance was negatively correlated with BMI (r = −0.74, *p* = 0.006), waist circumference (r = −0.62, *p* = 0.03), hip circumference (r = −0.80, *p* = 0.001), left arm circumference (r = −0.68, *p* = 0.002), bicipital skinfold thickness (r = −0.75, *p* = 0.02), subscapular skinfold thickness (r = −0.69, *p* = 0.01), and FM (r = −0.71, *p* = 0.008). This aligns with the recognized role of *Actinobacteria* in carbohydrate metabolism and SCFA production, which are vital for muscle function and energy homeostasis [54]. Their variable correlations across groups may reflect differential microbial resilience to dietary xenobiotics or fiber content, suggesting a regulatory function influenced by diet quality and composition.

The *Firmicutes Oscillospiraceae* family shows a strong and negative correlation with several anthropometric and body composition parameters, including BMI (*r* = −0.68, *p* = 0.01), left arm circumference (*r* = −0.70, *p* = 0.01), bicipital skinfold (*r* = −0.75, *p* = 0.004), tricipital skinfold (*r* = −0.70, *p* = 0.01), subscapular skinfold (*r* = −0.74, *p* = 0.008), and FM (*r* = −0.63, *p* = 0.02) in the IMOD. This is consistent with prior studies indicating that certain taxa within this family, such as *Oscillospira*, are more abundant in lean individuals and may be involved in SCFA production, particularly butyrate, which exerts anti-inflammatory effects and supports gut barrier integrity [55]. Interestingly, despite previous associations with constipation or slower intestinal transit, *Oscillospiraceae* have also been linked to high-fiber diets and metabolic resilience. In our study, *Oscillospiraceae* emerged as a key taxon discriminating the IMOD from IMnOD groups, being significantly more abundant in the former. This underscores the potential of organic dietary patterns to modulate GM favorably, promoting taxa associated with improved metabolic health.

Conversely, the *Bacteroidaceae* family and *Bacteroides* genus were primarily associated with higher FM% (*r* = 0.50, *p* = 0.02) in the No Diet group. This observation is consistent with studies indicating that diets rich in saturated fats and low in fiber favor the proliferation of *Bacteroides* spp. [56], which have been linked to metabolic endotoxemia and dysbiosis [57]. Additionally, the genus *Butyricimonas*, belonging to the family *Odoribacteraceae*, was found in higher abundance in the No Diet group compared to both the IMOD and IMnOD groups. While *Butyricimonas* is known for SCFA production, its elevated presence in the absence of structured dietary intervention may reflect a compensatory microbial response to unbalanced dietary intake [58,59].

Taken together, these results support the notion that integrating organic food into a Mediterranean dietary pattern may enhance its functional impact on human health, particularly through the modulation of the gut ecosystem.

A major strength of this study lies in its originality. To the best of our knowledge, this is the first human intervention trial to investigate the comparative effects of a IMOD, IMnOD, and a No Diet on GM composition. While the health benefits of the Mediterranean Diet have been extensively documented, the specific contribution of food origin, namely organic versus conventional, remains largely unexplored in the context of microbial ecology. By addressing this gap, our study offers novel insights into how dietary quality, beyond macronutrient content, may influence host–microbiota interactions. Importantly, both dietary interventions were matched for caloric content and macronutrient distribution, thereby isolating the variable of food origin as the principal discriminant. The use of validated tools such as the MEDAS and FFQ questionnaires, alongside anthropometric and bioimpedance measurements, further enriched the multidimensional characterization of the intervention’s effects.

Nevertheless, certain limitations must be acknowledged. The relatively small sample size, while appropriate for within-subject microbial comparisons, limits the generalizability of the results and precludes the performance of subgroup analyses based on age and sex. This is a critical consideration, as emerging evidence indicates that hormonal, immunological, and metabolic differences between men and women can significantly shape the composition and function of the gut microbiota, potentially leading to sex-specific responses to dietary interventions such as IMOD. Accordingly, future studies should adopt stratified designs or larger cohorts to systematically explore these interactions. Additionally, the duration of each dietary intervention was limited to four weeks. Although enough to elicit detectable changes in microbial community structure, longer intervention periods might be necessary to capture sustained functional outcomes or metabolic adaptations. Another limitation lies in the analytic approach: 16S rRNA gene sequencing allowed for taxonomic profiling but did not provide functional or metabolomic insights. Advanced omics methodologies, including metagenomics, metatranscriptomics, and targeted metabolomics, would be necessary to elucidate the host–microbe metabolic crosstalk and validate functional implications. Finally, the unblinded design of the intervention, inherent to dietary trials, may have introduced bias in terms of participant compliance or self-reported dietary data, despite the application of standardized protocols and objective biomarkers.

While this study is strengthened by its innovative design, it also highlights important areas for further research. The data presented supports the hypothesis that organic food quality can enhance the microbiota-modulatory effects of the Mediterranean Diet, but larger, longer-term studies with integrative multi-omics approaches are needed to confirm and extend these findings.

## 5. Conclusions

This randomized controlled pilot study provides novel evidence that not only the overall structure of the Mediterranean Diet, but also the origin and quality of its components, significantly shape GM composition. Transitioning from a habitual unrestricted diet to a structured Mediterranean dietary pattern led to favorable microbial shifts, particularly through the enrichment of taxa previously associated with SCFA production and intestinal health.

No major changes were observed in anthropometric or body composition parameters over the short intervention period. Importantly, while both Mediterranean diets induced microbiota changes, no consistent evidence emerged to support a globally stronger effect of the IMOD over IMnOD. Nonetheless, specific taxa such as *Parabacteroides distasonis* and *Anaerostipes hadrus* showed greater increases in IMOD, potentially reflecting subtle differences in microbial responsiveness to organic food intake.

Additionally, sex emerged as a relevant factor influencing microbial composition, with distinct taxa contributing to the separation between male and female GM profiles. Although this study was not powered for subgroup analyses, these findings underscore the importance of integrating sex-specific considerations into microbiome research.

Overall, our results highlight the potential for Mediterranean dietary interventions, especially with organic food (IMOD), to modulate the ecology of the gut microbiota and support host health. Further research with larger sample sizes, longer follow-up periods, and integrated multi-omics analyses is warranted to confirm these findings and explore their long-term clinical relevance.

## Figures and Tables

**Table 1:** Baseline characteristics of the study population.

Characteristics	
Sample size	39 (100)
Females	27 (69.23)
Males	12 (30.77)
Age (years)	38.46 ± 10.35
Height (cm)	166.56 ± 7.10
Weight (kg)	67.12 ± 14.37
BMI (kg/m^2^)	24.03 ± 3.93

Values are expressed as mean and standard deviation (M ± SD) for continuous variables or as number and percentage (*n* (%)) for categorical variables. Abbreviations: BMI, body mass index.

**Table 2:** Comparison of IMOD and IMnOD nutritional characteristics.

	IMOD	IMnOD	*p*-Value
Kcal	2087.00 ± 12.72	2069.00 ± 12.72	0.31
Proteins (%)	20.65 ± 0.07	20.55 ± 0.07	0.31
Lipids (%)	34.85 ± 0.21	34.55 ± 0.21	10.00
Carbohydrates (%)	44.72 ± 0.10	44.57 ± 0.10	0.31
Proteins (g)	103.32 ± 0.38	102.77 ± 0.38	0.31
Lipids (g)	77.55 ± 0.62	76.66 ± 0.62	0.31
Carbohydrates (g)	239.29 ± 2.26	236.09 ± 2.26	0.31
Total Fibers (g)	42.77 ± 1.30	40.92 ± 1.30	0.31
Cholesterol (mg)	209.50 ± 2.12	206.50 ± 2.12	0.31
Saturated Fatty Acids (g)	18.415 ± 0.01	18.40 ± 0.01	0.31
Saturated Fatty Acids (%/kcal tot)	8.31 ± 0.10	8.17 ± 0.10	0.31
Salt (g)	3.85 ± 0.07	3.75 ± 0.07	0.31
Sodium (mg)	1561.38 ± 25.48	1525.34 ± 25.48	0.31
Glycemic Index	57.98 ± 0.18	57.72 ± 0.18	0.31
ω3	3.65 ± 0.21	3.35 ± 0.21	0.31
ω6	10.22 ± 0.10	10.07 ± 0.10	0.31
ω6/ω3	3.02 ± 0.14	2.81 ± 0.14	0.31
ORAC	19,882.50 ± 976.51	18,501.50 ± 976.51	0.31
PRAL	−1.93 ± 4.22	−7.91 ± 4.22	0.31
MAI	15.05 ± 0.07	14.95 ± 0.07	10.00
AI	0.17 ± 0.01	0.16 ± 0.01	0.31
TI	0.22 ± 0.01	0.20 ± 0.01	0.31

Values are expressed as mean and standard deviation (M ± SD) for continuous variables. Nonparametric Wilcoxon analysis was performed. *p* < 0.05 indicates statistical significance. Abbreviations: AI, Atherogenic Index; g, grams; Kcal, Kilocalories; MAI, Mediterranean Adequacy Index; mg, milligrams; ORAC, Oxygen Radical Absorbance Capacity; PRAL, Potential Renal Acid Load; TI, Thrombogenic Index; ω, omega fatty acids.

**Table 3:** Comparison of participants’ body composition characteristics between groups.

Variables	No Diet (*n* = 13)	IMOD (*n* = 13)	IMnOD (*n* = 13)	*p*-Value
Females	9	9	9	-
Males	4	4	4	-
Weight (kg)	67.12 ± 15.21	66.03 ± 15.20	65.54 ± 15.22	0.88
BMI (kg/m^2^)	24.03 ± 4.04	23.53 ± 3.99	23.68 ± 4.02	0.79
Neck circumference (cm)	33.62 ± 3.70	32.82 ± 3.12	32.96 ± 3.51	0.78
Waist circumference (cm)	75.62 ± 11.12	74.74 ± 11.48	74.11 ± 9.92	0.84
Abdomen circumference (cm)	84.97 ± 10.46	82.08 ± 10.62	84.09 ± 10.69	0.49
Hip circumference (cm)	97.26 ± 6.96	95.96 ± 6.66	96.83 ± 6.92	0.63
WHR	0.78 ± 0.09	0.78 ± 0.09	0.76 ± 0.07	0.99
Wrist circumference (cm)	15.37 ± 1.16	15.14 ± 1.05	15.17 ± 1.17	0.61
Bicipital Fold (mm)	9.00 ± 4.82	6.21 ± 3.84	8.63 ± 4.44	0.13
Tricipital Fold (mm)	18.67 ± 8.80	14.42 ± 7.38	12.92 ± 5.68	0.21
Subscapular fold (mm)	16.08 ± 9.35	9.55 ± 4.93	12.13 ± 5.69	0.05
Suprailiac fold (mm)	14.67 ± 11.14	11.38 ± 5.75	10.29 ± 4.81	0.38
Mean arms circumference (cm)	27.97 ± 3.81	27.30 ± 3.47	27.71 ± 3.39	0.65
Mean mid thighs circumference (cm)	50.21 ± 4.67	50.63 ± 3.83	50.73 ± 4.06	0.81
Mean root thighs circumference (cm)	57.00 ± 4.82	57.13 ± 3.80	57.53 ± 4.39	0.94
BAI (%)	27.33 ± 3.48	26.55 ± 3.01	27.43 ± 3.20	0.54

Values are expressed as mean and standard deviation (M ± SD) for continuous variables. ANOVA analysis was performed. *p* < 0.05 indicates statistical significance. Abbreviations: BAI, body adiposity index; BMI, body mass index; IMnOD, Italian Mediterranean non-Organic Diet; IMOD, Italian Mediterranean Organic Diet; No Diet: free diet; WHR, waist hip ratio.

**Table 4:** Comparison of participants’ BIA parameters between groups.

Variables	No Diet (*n* = 13)	IMOD (*n* = 13)	IMnOD (*n* = 13)	*p*-Value
Rz (ohm)	577.00 ± 78.50	581.67 ± 83.15	585.91 ± 84.75	0.92
Xc (ohm)	60.62 ± 8.33	60.81 ± 10.56	61.12 ± 10.05	0.98
PhA (°)	6.05 ± 0.60	6.00 ± 0.74	6.01 ± 0.78	0.96
Z (ohm)	580.20 ± 78.71	584.88 ± 83.48	589.14 ± 84.97	0.91
FM (kg)	18.98 ± 8.26	17.72 ± 7.83	17.87 ± 8.25	0.81
FM (%)	25.72 ± 7.89	26.08 ± 8.22	26.79 ± 8.56	0.88
FFM (kg)	48.96 ± 11.64	48.55 ± 11.31	47.55 ± 10.87	0.88
FFM (%)	73.38 ± 7.84	73.92 ± 8.22	73.21 ± 8.56	0.94
TBW (L)	34.92 ± 10.34	35.08 ± 9.86	33.58 ± 10.78	0.83
TBW (%)	48.92 ± 6.07	48.83 ± 8.00	49.04 ± 6.40	0.99
ECW (L)	17.65 ± 8.15	15.50 ± 3.47	15.33 ± 3.29	0.21
ECW (%)	44.90 ± 9.00	46.87 ± 3.06	46.98 ± 2.90	0.31
ICW (L)	20.67 ± 12.20	17.77 ± 4.82	17.46 ± 4.59	0.26
ICW (%)	50.37 ± 8.79	53.13 ± 3.06	53.04 ± 2.90	0.12
BCM (kg)	28.48 ± 9.27	26.85 ± 6.48	27.78 ± 8.13	0.74
BCM (%)	39.54 ± 4.39	40.86 ± 5.58	40.37 ± 7.91	0.71
BCMI (kg/m^2^)	10.10 ± 2.36	9.53 ± 1.56	9.96 ± 2.09	0.54

Values are expressed as mean and standard deviation (M ± SD) for continuous variables. ANOVA analysis was performed. *p* < 0.05 indicates statistical significance. Abbreviations: BCM, Body Cell Mass; BCMI, Body Cell Mass Index; ECW, Extracellular Water; ICW, Intracellular Water; IMnOD, Italian Mediterranean non-Organic Diet; IMOD, Italian Mediterranean Organic Diet; No Diet: free diet; FFM, Fat Free Mass; FM, Fat Mass; PhA, Angle Phase; Rz, Resistance; TBW, Total Body Water; Xc, Reactance; Z, Impedance.

**Table 5:** Top 30 microbial taxa discriminating between the ΔNo diet, ΔIMOD, and ΔIMnOD groups, as identified by sparse Partial Least Squares Discriminant Analysis (sPLS-DA).

Discriminative Microbial Features	Loading 1	Loading 2	ΔIMOD	ΔIMnOD	ΔNoDiet	*p*-Value	FDR
*Blautia luti*	−0.49	-	3.08	6.86	0.01	0.06	0.21
*Veillonella tobetsuensis*	−0.44	-	0.00	0.01	0.00	0.01	0.08 *
*Collinsella aerofaciens*	−0.43	-	0.89	2.41	0.01	0.01	0.08 *
*Agathobaculum desmolans*	−0.37	-	0.01	0.01	0.00	0.01	0.08 *
*Bacteroides uniformis*	0.24	-	0.46	−0.83	−0.01	0.02	0.12
*Merdimonas faecis*	−0.17	-	0.00	0.01	0.00	0.04	0.18
*Anaerobium acetethylicum*	−0.14	-	0.00	0.01	0.00	0.04	0.18
*Anaerobutyricum hallii*	−0.12	−0.02	1.16	1.47	0.01	0.01	0.08 *
*Lactonifactor longoviformis*	−0.10	-	0.00	0.01	0.00	0.12	0.21
*Bifidobacterium catenulatum*	−0.10	-	0.00	0.06	0.00	0.12	0.21
*Slackia isoflavoniconvertens*	−0.09	-	−0.01	0.04	−0.01	0.73	0.75
*Blautia schinkii*	−0.09	-	−0.01	0.11	−0.01	0.11	0.21
*Streptococcus saliviloxodontae*	−0.09	-	0.00	0.01	0.00	0.12	0.21
*Anaerotignum aminivorans*	−0.09	-	0.00	0.01	0.00	0.12	0.21
*Peptacetobacter hiranonis*	−0.09	-	0.00	0.01	0.00	0.12	0.21
*Streptococcus koreensis*	−0.09	-	0.00	0.04	0.00	0.12	0.21
*Bifidobacterium pseudolongum*	−0.08	-	0.00	0.01	0.00	0.12	0.21
*Bifidobacterium adolescentis*	−0.07	-	0.22	1.01	0.00	0.61	0.65
*Lachnospira eligens*	0.06	-	−0.26	−0.64	−0.01	0.85	0.86
*Bifidobacterium choerinum*	−0.06	-	0.00	0.01	0.00	0.12	0.21
*Romboutsia timonensis*	−0.05	-	0.95	1.18	−0.01	0.01	0.01 *
*Holdemanella biformis*	−0.05	-	0.08	0.61	−0.01	0.65	0.69
*Parabacteroides distasonis*	0.04	-	0.15	−0.60	0.01	0.01	0.08 *
*Defluviitalea saccharophila*	−0.04	-	−0.01	0.01	0.01	0.07	0.21
*Gabonia massiliensis*	0.04	-	−0.02	−0.04	0.01	0.08	0.21
*Dorea longicatena*	−0.02	-	0.97	1.61	−0.01	0.01	0.08 *
*Bifidobacterium longum*	−0.01	-	0.49	1.86	−0.01	0.48	0.56
*Streptococcus sanguinis*	−0.01	-	0.00	0.01	0.00	0.12	0.21
*Streptococcus mitis*	−0.01	-	0.01	0.04	−0.01	0.49	0.56
*Solibacillus isronensis*	−0.01	-	0.01	0.03	−0.01	0.16	0.24
*Anaerostipes hadrus*	-	−0.63	2.69	1.18	−0.01	0.01	0.01 *
*Erysipelatoclostridium ramosum*	-	−0.40	0.31	0.01	−0.01	0.02	0.12
*Arthrobacter citreus*	-	−0.35	0.44	0.05	0.01	0.16	0.24
*Bifidobacterium pseudocatenulatum*	-	−0.30	0.91	0.33	0.01	0.26	0.36
*Escherichia coli*	-	−0.21	0.42	−0.05	0.01	0.21	0.31
*Lacrimispora saccharolytica*	-	−0.15	0.16	0.03	0.00	0.06	0.21
*Flavonifractor plautii*	-	−0.15	0.68	0.11	0.01	0.17	0.24
*Megasphaera indica*	-	−0.13	0.46	0.03	−0.01	0.07	0.21
*Prevotella disiens*	-	−0.13	0.02	0.01	0.00	0.32	0.41
*Phocaeicola coprocola*	-	−0.13	0.84	−0.01	−0.01	0.61	0.65
*Desulfovibrio simplex*	-	−0.09	0.23	−0.01	−0.01	0.49	0.56
*Clostridium saudiense*	-	−0.08	0.85	0.46	−0.01	0.01	0.08 *
*Flintibacter butyricus*	-	−0.08	0.25	−0.03	0.01	0.16	0.24
*Peptoniphilus asaccharolyticus*	-	−0.07	0.06	0.01	0.00	0.32	0.41
*Granulicatella adiacens*	-	−0.07	0.01	0.01	−0.01	0.28	0.37
*Anaerotruncus rubiinfantis*	-	−0.07	0.14	0.04	0.01	0.94	0.94
*Enterococcus hermanniensis*	-	−0.07	0.65	0.00	0.00	0.12	0.21
*Anaerostipes butyraticus*	-	−0.07	0.51	−0.04	−0.01	0.04	0.18
*Streptococcus lactarius*	-	−0.05	0.12	0.00	0.00	0.12	0.21
*Clostridium saccharogumia*	-	−0.05	0.12	−0.05	−0.01	0.11	0.21
*Clostridium Disporicum*	-	−0.05	0.26	0.00	0.00	0.12	0.21
*Dialister succinatiphilus*	-	0.04	−0.59	−0.21	−0.01	0.41	0.51
*Eubacterium eligens*	-	−0.04	0.85	−0.03	−0.01	0.49	0.56
*Actinomyces odontolyticus*	-	−0.03	0.03	0.00	0.00	0.12	0.21
*Ruminococcus lactaris*	-	−0.03	0.30	−0.03	−0.01	0.54	0.61
*Anaerovibrio lipolyticus*	-	−0.02	0.12	0.00	0.00	0.12	0.21
*Butyricimonas synergistica*	-	−0.01	0.06	0.00	0.00	0.12	0.21
*Clostridium jeddahense*	-	−0.01	0.01	−0.01	−0.01	0.27	0.36
*Duodenibacillus massiliensis*	-	0.01	−0.04	−0.01	0.01	0.08	0.21

The table shows the taxonomic classification of each feature and its relative contribution to group separation (FDR). Those with FDR < 0.1 are especially considered significant contributors to group separation. Moreover, we reported the Δ concentration of each taxa in different groups. “Loading 1” and “Loading 2” refer to the contributions of each taxon to the corresponding latent components identified by sPLS-DA. Positive and negative values indicate the direction of association with the discriminant axes and do not directly reflect increased or decreased abundance. Taxa with negative loadings are associated with the opposite side of the component axis compared to those with positive loadings, thus contributing to group separation in the opposite direction. Abbreviations: IMnOD, Italian Mediterranean non-Organic Diet; IMOD, Italian Mediterranean Organic Diet; No Diet: free diet. * *p* < 0.05 indicates statistical significance.

## Data Availability

The original contributions presented in this study are included in the article. Further inquiries can be directed to the corresponding author.

## Abbreviations

The following abbreviations are used in this manuscript:

3-PBA	3-Phenoxybenzoic Acid
ANOVA	Approximate One-Way Analysis Of Variance
BAI	Body Adiposity Index
BCM	Body Cell Mass
BCMI	Body Cell Mass Index
BIA	Bioimpedance Analysis
BMI	Body Mass Index
BMR	Basal Metabolic Rate
DAPs	Dialkylphosphates
ECM	Extracellular Mass
ECW	Extracellular Water
FFM	Fat Free Mass
FFQ	Food Frequency Questionnaire
FM	Fat Mass
GM	Gut Microbiota
ICW	Intracellular Water
IMnOD	Italian Mediterranean Non-Organic Diet
IMOD	Italian Mediterranean Organic Diet
MEDAS	Mediterranean Diet Adherence Screener
No Diet	Free Diet
OTUs	Operational Taxonomic Units
PCoA	Principal Coordinates Analysis
PERMANOVA	Permutational Multivariate Analysis Of Variance
PhA	Phase Angle
REML	Restricted Maximum Likelihood
Rz	Resistance
SCFAs	Short Chain Fatty Acids
SD	Standard Deviation
sPLS-DA	Partial Least Squares Discriminant Analysis
TBW	Total Body Water
WHO	World Health Organization
WHR	Waist-To-Height Ratio
Xc	Reactance
Z	Impedance

## Appendix A

Figure A1 reports the discriminant taxa associated with Component 1 and Component 2, obtained from sPLS-DA analysis to Δ data.

Figure A2, Figure A3, Figure A4 and Figure A5 report the discriminant taxa associated with Component 1 and Component 2, obtained from sPLS-DA analysis to Δ data.

Figure A6, Figure A7 and Figure A8 show heatmaps that illustrate the Pearson correlation coefficients (r) between anthropometric measures, dietary variables, and microbial taxa at the phylum and genus levels in the No diet, IMOD and IMnOD groups.
